# Modelling the impact of the Omicron BA.5 subvariant in New Zealand

**DOI:** 10.1098/rsif.2022.0698

**Published:** 2023-02-01

**Authors:** Audrey Lustig, Giorgia Vattiato, Oliver Maclaren, Leighton M. Watson, Samik Datta, Michael J. Plank

**Affiliations:** ^1^ Manaaki Whenua, Lincoln, New Zealand; ^2^ Mathematics and Statistics, University of Canterbury, Christchurch, New Zealand; ^3^ Department of Physics, The University of Auckland, Auckland, New Zealand; ^4^ Department of Engineering Science, University of Auckland, Auckland, New Zealand; ^5^ School of Earth and Environment, University of Canterbury, Christchurch, New Zealand; ^6^ National Institute of Water and Atmospheric Research, Wellington, New Zealand

**Keywords:** epidemiology, COVID-19, immunity, waning, approximate Bayesian computation, dynamic model

## Abstract

New Zealand experienced a wave of the Omicron variant of SARS-CoV-2 in early 2022, which occurred against a backdrop of high two-dose vaccination rates, ongoing roll-out of boosters and paediatric doses, and negligible levels of prior infection. New Omicron subvariants have subsequently emerged with a significant growth advantage over the previously dominant BA.2. We investigated a mathematical model that included waning of vaccine-derived and infection-derived immunity, as well as the impact of the BA.5 subvariant which began spreading in New Zealand in May 2022. The model was used to provide scenarios to the New Zealand Government with differing levels of BA.5 growth advantage, helping to inform policy response and healthcare system preparedness during the winter period. In all scenarios investigated, the projected peak in new infections during the BA.5 wave was smaller than in the first Omicron wave in March 2022. However, results indicated that the peak hospital occupancy was likely to be higher than in March 2022, primarily due to a shift in the age distribution of infections to older groups. We compare model results with subsequent epidemiological data and show that the model provided a good projection of cases, hospitalizations and deaths during the BA.5 wave.

## 1. Introduction

The B.1.1.529 (Omicron) variant of SARS-CoV-2 was designated a variant of concern by the World Health Organization on 26 November 2021 [1] subsequent to a rapid growth in cases of COVID-19 in southern Africa [2]. Following transmission from cases in managed isolation facilities into the community in January 2022 [3], New Zealand experienced a large Omicron wave with a cumulative attack rate of around 220 confirmed cases per 1000 people between 1 February and 31 May 2022. This wave was dominated by the BA.2 subvariant, which accounted for an estimated 84% of cases, with BA.1 accounting for the remaining 16%. In the week ending 29 May 2022, over 95% of sequenced new community cases were BA.2 [4].

Prior to the arrival of Omicron, the total number of confirmed community cases of COVID-19 in New Zealand was only around 0.25% of the population due to a combination of border restrictions, vaccination and strong public health measures [5]. This meant the population had very low levels of immunity from prior infection at this time. By 1 February 2022, 77% of the population (90% of those aged over 12 years) had received at least two vaccine doses and 27% of the population (35% of those aged over 18 years) had received a third dose. By 1 April 2022, third dose coverage had increased to 51% of the population (66% of those aged over 18 years). In addition, those aged 5–11 years became eligible for vaccination on 17 January 2022, and by 1 April, 54% of this age group had received at least one dose and 17% had received two doses. The Pfizer/BioNTech BNT162b2 vaccine was the main vaccine in use and accounted for the majority of all doses administered.

Public health measures to control the spread of the virus were applied as part of New Zealand’s COVID-19 Protection Framework [6]. The country was moved to the Red setting of the framework on 23 January 2022, which included widespread mask mandates, gathering size limits, vaccine pass requirements for many businesses and encouraged working from home. These restrictions were relaxed in stages with outdoor gathering restrictions removed and indoor gathering size limits increased from 100 to 200 on 25 March 2022. Vaccine pass requirements were removed on 4 April. The country was moved to the Orange setting on 13 April 2022, meaning that all gathering restrictions were lifted and masks were encouraged but no longer mandatory in schools. Isolation requirements were also progressively reduced from 14 days to 7 days, and quarantine requirements were narrowed to just household contacts on 12 March 2022.

The BA.5 subvariant was first detected in South Africa in February 2022 [7] and is closely related to BA.2. It carries distinct mutations in the spike protein, two of which are associated with immune evasion and higher transmissibility relative to the previously dominant BA.1 and BA.2 variants [8,9]. BA.5 has driven waves of COVID-19 in multiple countries [10]. The rise in BA.5 stems at least in part from its ability to infect people who were immune to earlier variants, but so far there is no indication the variant causes more severe disease. BA.5 was first detected in the New Zealand community in April 2022, and cases have been appearing consistently since May. It quickly rose to 32% of sequenced community cases by the beginning of July and became the dominant variant in early July 2022 [4].

Mathematical modelling has been a key tool informing the response to COVID-19 in countries around the world and has been used in various ways. In the early stages of the pandemic, modelling provided insights into the epidemiological characteristics of SARS-CoV-2 [11–13]. Models have been used for reproduction number estimation and short-term forecasting of the number of new cases [14,15], for projecting the likely impact of relaxation of non-pharmaceutical interventions [16] and for longer-term scenarios comparing alternative strategies or epidemiological outcomes [17–19]. Models have been deployed in real time to model new variants of concern, such as the Alpha, Delta and Omicron variants [20–22]. The aim of the modelling described in this study was to understand the likely impact of the BA.5 subvariant in New Zealand.

We have previously modelled the spread and impact of the Omicron variant in New Zealand using an age-structured stochastic model [23]. This model included the effects of age-specific vaccination rates, different vaccine effectiveness against different clinical endpoints and waning of vaccine-derived immunity. The model results were reasonably consistent with the observed numbers of cases, hospitalizations and deaths during the first Omicron wave up to early April 2022.

Here, we generalize this model to include waning of infection-derived immunity, meaning that people can be reinfected with the virus for a second time. Due to the high prevalence of cases in the community during 2022, we switch from using a stochastic to deterministic model, which significantly increases computational efficiency with a minimal impact on the overall epidemic dynamics. We also develop a novel model component for the displacement of the resident variant by a new variant with different transmissibility and immune escape characteristics. We calibrate model parameters for the new variant using estimates of the growth advantage of BA.5 relative to BA.2 from data on whole genome sequencing of community cases [24]. We fit the model to New Zealand epidemiological data up to early July 2022 and use the model to estimate the future impact of the BA.5 subvariant under different levels of immune escape. These results were used in real time to inform New Zealand government policy and healthcare system preparedness in advance of and during the wave. Here, we compare the model projections with subsequent data on new daily cases, hospital admissions and deaths.

## 2. Methods

**Figure 1.**
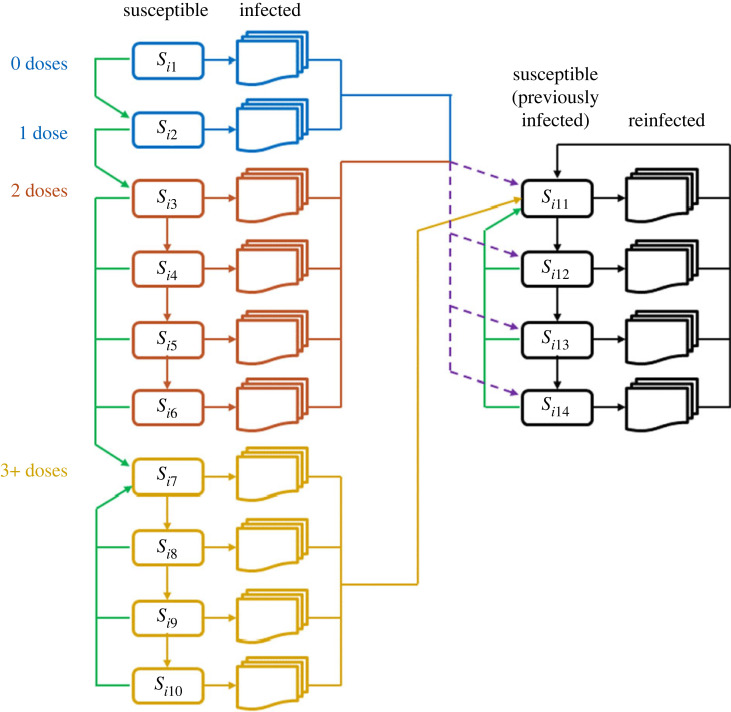
Schematic diagram of the model structure showing the 14 susceptible compartments for age group *i*, indexed as compartments *S*_*ik*_ for *k* = 1, …, 14. Vertical downward arrows represent transition to a susceptible compartment with lower immunity as a result of waning immunity. Green arrows represent transition to a susceptible compartment with higher immunity as a result of vaccination. Horizontal arrows represent infection, which initiates transition through a series of disease states ending in recovery. Following recovery from first infection, individuals who have had at least three vaccine doses (yellow) transition to the highest immunity post-infection compartment *S*_*i*,11_; individuals who have had fewer than three vaccine doses (blue and red) transition to a mixture of compartments *S*_*i*,11_ to *S*_*i*,14_ (dashed purple arrows), representing lower post-infection immunity for these groups. Following recovery from a second or subsequent infection (black), all individuals transition to *S*_*i*,11_ regardless of vaccination status.

The susceptible population is divided into *n*_*A*_ = 16 age groups and *n*_*S*_ = 14 susceptible compartments per age group, denoted *S*_*ik*_ for *i* = 1, …, *n*_*A*_ and *k* = 1, …, *n*_*S*_. The susceptible states represent different levels of vaccine-derived and infection-derived immunity (figure 1). Each state *k* is associated with a set of immunity parameters *e*_*Ok*_ representing immunity against different outcomes *O* (see §2.1). Transmission between age groups is governed by a contact matrix [23,25]. See electronic supplementary material, section S1.1 for details of the ordinary differential equations governing transmission dynamics. Matlab code to run the model and reproduce the results is available at https://github.com/michaelplanknz/modelling-ba5-in-nz.

The model structure is similar to that of the stochastic model of Vattiato *et al.* [23] but is generalized to include waning of infection-derived immunity and the effects of fourth and potentially subsequent doses of the vaccine. Using a deterministic model ignores stochastic fluctuations in daily infection rates, although this is likely to have a relatively small effect on epidemic dynamics during periods of relatively high prevalence. The waning model is similar to the study by Keeling *et al.* [22], but the inclusion of a series of post-recovery susceptible compartments means the model is not restricted to exponential waning curves and can capture differing dynamics of immunity against infection and immunity against severe disease. The immunity model is age independent.

Vaccination rates are based on the number of vaccine doses per day given to people in age group *i* at time *t*, plus estimated future uptake of fourth doses according to Ministry of Health projections (see electronic supplementary material, figure S2). Case reporting, hospital admissions and discharges and deaths are modelled via age-dependent rates of clinical disease, hospitalization and fatality, and probability of testing (see electronic supplementary material, section S1.5 and tables S1 and S2).

### 2.1. Immunity model

The model includes parameters representing the level of immunity against infection (*e*_*I*,*k*_), symptomatic disease (*e*_*S*,*k*_), transmission (*e*_*T*,*k*_), hospitalization (*e*_*H*,*k*_) and death (*e*_*F*,*k*_) for people in susceptible compartment *k*. In principle, this means that there are a total of up to 70 age-independent immunity parameters in the model (14 susceptible compartments times five endpoints). To provide a parsimonious parametrization, we use the model of Khoury *et al.* [26] and Cromer *et al.* [27] for the relationship between the level of immunity and neutralizing antibody titre. This approach to parametrizing models has also been used for other groups carrying out epidemiological modelling for policy advice [20]. The antibody titre is assumed to be a correlate of protection, and a given titre is generally more protective against more serious clinical endpoints, in line with the findings of Cromer *et al.* [28]. This framework enables laboratory data from neutralization experiments to be combined with population-level data to produce estimates of time-varying immunity from different sources, to different endpoints, resulting from infection with different variants of SARS-CoV-2 [29].

We use the estimates of Golding and Lydeamore [29] to determine the initial log antibody titre *n*_*l*,0_ associated with each source of immunity *l* in our model (two or three vaccine doses with or without prior Omicron infection). To model waning immunity, we assume that the log antibody titre decreases by a fixed amount for each successive susceptible compartment in the same category (i.e. through compartments *k* = 3, …, 6, *k* = 7, …, 10 and *k* = 11, …, 14). We then map the log antibody titre *n*_*k*_ for compartment *k* to immunity *e*_*Ok*_ against outcome *O* via a logistic function with an outcome-specific midpoint parameter *n*_*O*,50_ [26],

2.1 $$e_{Ok}=\frac{1}{1+e^{-\kappa (n_{k}-n_{O,50})}}.$$

This framework means the immunity model can be parametrized with one parameter *n*_*l*,0_ for each source of immunity *l*, one parameter for each outcome *O* and two additional independent parameters: the logistic slope *κ* and the transition rate *r*_*w*_ between successive susceptible compartments, which represents the speed of waning (see electronic supplementary material, table S3).

For the post-infection susceptible states, we do not have separate sets of susceptible compartments for people with different vaccination status. Instead, we model vaccination-dependent levels of post-infection immunity by moving people to different susceptible compartments dependent on their vaccination status. Following recovery from a first infection, people who have had three doses of the vaccine all move initially to the highest immunity compartment *k* = 11. People who have had fewer than three doses of the vaccine move to the lower-immunity compartments *k* = 12, 13, 14 in specified proportions (dashed purple arrows in figure 1; see electronic supplementary material, section S1.4). We assume that, following recovery from a second or subsequent infection, everyone moves to the highest immunity compartment *k* = 11 regardless of vaccination status. We assume that a fourth (or subsequent) dose of the vaccine prior to infection restores people to the same immunity level (susceptible compartment *k* = 7) as immediately after the third dose, and that any dose of the vaccine after recovery from infection moves people to the highest immunity level (susceptible compartment *k* = 11). This is a model simplification to avoid having to keep track of too many different vaccination and infection histories rather than having an epidemiological rationale (see also §4).

For simplicity, we set *e*_*Tk*_ = 0 and *e*_*Sk*_ = *e*_*Ik*_, i.e. we assume that immunity reduces the risk of infection but, conditional on infection, does not change the likelihood of symptomatic disease or transmission. We also assume that immunity against hospitalization and death never wane below *e*_sev_, min = 0.5. This models a more durable component of the immune response, e.g. cellular immunity as opposed to neutralizing antibodies, that maintains immunity against severe disease at some minimum long-term level. For the initial log titre following infection, we use somewhat higher values than those estimated by Golding and Lydeamore [29] (see electronic supplementary material, table S3). This is partly due to recent epidemiological studies, suggesting that prior infection with BA.1/BA.2 provides relatively strong immunity against reinfection with the same subvariant or a different Omicron subvariant, at least for several months [30–32]; and partly due to empirical observations that the proportion of new cases in New Zealand that were potential reinfections (had a previously reported positive test result at least 28 days prior) was relatively low (below 4% up to the end of June 2022—see §4).

**Figure 2.**
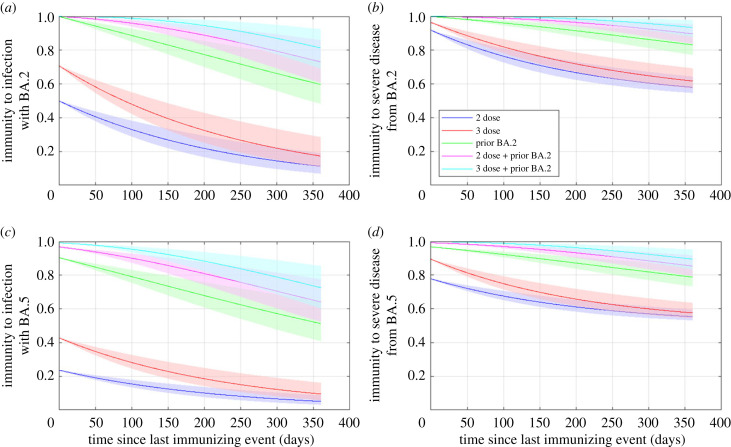
Average immunity under assumed parameter priors against: (*a*) infection with BA.2, (*b*) severe disease or death from BA.2, (*c*) infection with BA.5 and (*d*) severe disease or death from BA.5 as a function of time since most recent immunizing event. Graphs show immunity following two doses (blue), three doses (red), 0/1 doses and prior infection with BA.2 (yellow), two doses and prior infection with BA.2 (purple) and three doses and prior infection with BA.2 (green). Immunity from two or more prior Omicron infections also follows the green trajectory, regardless of the vaccination status. Immunity against BA.5 derived from prior infection with BA.5 is assumed to follow the same curves as for immunity against BA.2 derived from prior infection with BA.2. Curves are the median and shaded areas are the 5th and 95th percentiles of 500 random draws from the prior.

The average immunity from different sources against infection and against severe disease under assumed priors are shown in figure 2. At the time this modelling was carried out, data from epidemiological studies on the time dependence of immunity from prior Omicron infection against reinfection with BA.2 or BA.5 were scarce, and the picture was still rapidly evolving. The analysis of Golding and Lydeamore [29] that was used to parametrize these curves used published data from a range of epidemiological studies of vaccine effectiveness and laboratory studies on neutralizing antibody titres. Immunity to reinfection with BA.5 was still particularly uncertain. However, these priors are broadly consistent with available estimates of the strength of infection-derived immunity after approximately six months in highly vaccinated populations. Altarawneh *et al.* [30] estimated that previous Omicron infection was 78.0% (95% confidence interval (CI), 75.0–80.7%) protective against reinfection with BA.5 in Qatar. Malato *et al.* [32] produced a comparable estimate of 75.3% (95% CI, 75.0–75.6%) in Portugal. Hansen *et al.* [31] estimated the protection against BA.5 reinfection to be 92.7% (95% CI, 91.6–93.7%) and against severe disease to be 96.4% (95% CI, 74.2–99.5%) in Denmark.

Immunity from infections that occurred prior to the model seeding date in January 2022 is ignored. This is reasonable as there had only been around 2.5 confirmed community cases per 1000 people up to the end of 2021 [23]. Although no representative seroprevalence data are available for New Zealand to estimate the true number of infections, the public health strategy of intensive testing, case finding, contact tracing and source investigation meant that case ascertainment up to the end of 2021 was probably relatively high. Even if the ascertainment rate during this period were as low as 25%, that would mean that only around 1% of the population had been infected prior to the arrival of Omicron, and the impact of this on epidemic dynamics during the Omicron period would still be negligible. To ensure the model correctly captured waning of vaccine-derived immunity before the start of the first Omicron wave, we ran the model from a start date of 5 March 2021 (the beginning of the vaccine roll out in New Zealand) with vaccinations administered as per Ministry of Health data, but with no infections prior to the seeding date in January 2022.

#### 2.1.1. Variant model

To model the effect of a new variant of concern (VOC), we use a simplified approach that can capture potential changes in intrinsic transmissibility and/or immune escape. This does not encompass the full dynamics of two or more variants spreading simultaneously with partial cross-immunity [33], but captures the key effects by changing relevant model parameters around a specified time point *t*_*VOC*_ when the new variant becomes dominant. For simplicity, we assume that all infections before *t*_VOC_ are the resident variant (BA.2) and all infections after *t*_VOC_ are the new variant (BA.5), where *t*_VOC_ was set to 20 June 2022.

A variant that has different intrinsic transmissibility can be modelled by a change in the parameter *R*_EI_(*t*) at *t* = *t*_VOC_. A variant that evades immunity can be modelled by reducing the initial antibody titre levels *e*_2*d*,0_ and *e*_3*d*,0_ for vaccinated but not previously infected states at *t* = *t*_VOC_ [26]. This is equivalent to a reduction in vaccine effectiveness. We assume that BA.5 has the same intrinsic transmissibility as BA.2, but there is a 2.5-fold drop in antibody titre against BA.5 relative to BA.2, which is consistent with lab studies on neutralization [34,35].

Reducing the initial antibody titre for previously infected states (*k* = 11, …, 14) would result in a permanent reduction in infected-induced immunity, including against future reinfection with the same variant. To avoid this, we instead model reduction in infection-derived antibody titre to the new variant by moving individuals in the previously infected states (k=11,12 or 13) at *t* = *t*_VOC_ to a lower immunity state (k=12,13 or 14). This means that a reduction in average titre is applied to people infected before *t* = *t*_VOC_ (assumed to be infection with the resident variant), but people infected after *t* = *t*_VOC_ (assumed to be infection with the new variant) start with the same initial antibody titre following recovery as before the new variant arrived. Thus, the model assumes an equally high level of homologous immunity against reinfection with the same variant (whether resident→resident or VOC → VOC) but a relatively lower level of cross-reactive immunity to the new variant (resident → VOC). The loss of immunity due to the new variant occurs in the model via the same mechanism as loss of immunity due to waning, namely, transition of susceptibles to compartments with lower levels of immunity. The difference is that waning occurs gradually and continuously through time, whereas the variant effect occurs as a rapid transition to lower immunity compartments over a short period of time.

The magnitude of the drop in infection-derived immunity to the new variant is determined by a dimensionless parameter *r*_VOC_ (see electronic supplementary material, section S1.4). In practice, the value of *r*_VOC_ was manually tuned such that the change in the epidemic growth rate at time *t* = *t*_VOC_ corresponds to the empirically observed growth advantage of the new variant relative to the resident variant. The growth rate of BA.5 (new variant) relative to BA.2 (resident variant) in genomically sequenced New Zealand community cases reported up to 21 June 2022 [24] was estimated to be $0.10\;{\mathrm{day}}^{-1}$ (95% CI $0.073\text{-}0.128\;{\mathrm{day}}^{-1}$) via multi-nominal regression (see electronic supplementary material, figure S3). This is consistent with international estimates of the growth advantage of BA.5 over BA.2, which are generally in the range 0.07 to $0.14\;{\mathrm{day}}^{-1}$ [7,10]. Values of *r*_VOC_ = 0.39 ± 0.2 were found to produce an increase in the epidemic growth rate consistent with these estimates. Due to a lack of evidence to the contrary, we assumed that there is no change in disease severity for BA.5 compared with BA.2.

#### 2.1.2. Parameter inference and model fitting

We take a simple approximate Bayesian computation (ABC rejection) approach to fit the model to data. For each combination of parameter values drawn from the prior, we solve the model and calculate the error function *d*(*x*, *y*), where *x* is the time series of model outputs for a specified variable and *y* is the corresponding data time series. The 1% of model simulations with the smallest values of the error function is retained. We fit the following model outputs to data: (i) new daily cases, (ii) proportion of new cases in over-60s, (iii) new daily hospital admissions, (iv) daily deaths, and (v) new weekly infections. Outputs (i)–(iv) are fitted to official data reported by the New Zealand Ministry of Health; output (v) is fitted to data on the weekly incidence of new cases in a routinely tested cohort of approximately 20 000 border works (see electronic supplementary material, section S1.5 for details). The dataset used for fitting is available at https://github.com/michaelplanknz/modelling-ba5-in-nz.

Prior distributions for fitted parameters are shown in electronic supplementary material, tables S1–S3. Generally, these are relatively informative priors that represent modest uncertainty in parameter estimates. These include changes in the value of the reproduction number excluding immunity *R*_EI_(*t*) and contact matrix *M* during specified time windows to model changes in mixing rates as a result of public health interventions or voluntary behavioural change. The value of *R*_EI_(*t*) was assumed to increase linearly from *R*_EI,1_ to *R*_EI,2_ starting around 10 March 2022 and over a window of 35–75 days (see electronic supplementary material, table S1). The contact matrix *M* was initially set to the matrix in the study by Vattiato *et al.* [36], denoted *M*_0_, to provide a reasonable match with the observed age distribution of cases in the first part of the simulated time period. The contact matrix *M* was assumed to change to a modified matrix (1 − *α*_*M*_)*M*_0_ + *α*_*M*_ *M*_1_, where *M*_1_ is the matrix estimated from pre-pandemic data [25,37] and *α*_*M*_ ∈ [0, 1] is fitted to data (see electronic supplementary material, figure S1). The change in contact matrix was assumed to occur linearly over a 50–90 day time window starting at the same time as the change in *R*_EI_(*t*) (see electronic supplementary material, table S1). These are ad hoc model adjustments which were observed to provide a reasonable fit to data and reflect plausible behavioural changes during the simulated time period. Time windows are fitted to the data using the ABC algorithm.

## 3. Results

Results were fitted to data available on 7 July 2022 on new daily cases, hospitalizations and deaths, the proportion of new cases in over-60-year-olds, and new weekly infections in a cohort of routinely tested border workers. Note that hospitalizations exclude those who are not being treated primarily for COVID-19 and deaths exclude those where the cause of death is classified as unrelated to COVID-19 by the Ministry of Health. To allow for reporting lags, the most recent 40 days of admissions data (admissions after 28 May 2022) and 10 days of deaths data (deaths after 27 June 2022) were excluded. We then compare model projections with subsequent data, available on 22 September 2022.

**Figure 3.**
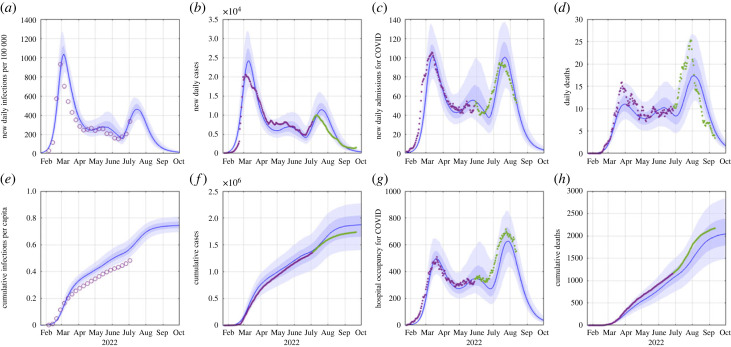
Model results showing: (*a*) daily infections per 100 000 people, (*b*) daily reported cases, (*c*) new daily hospital admissions for COVID-19, (*d*) daily COVID-19 deaths, (*e*) cumulative infections, (*f*) cumulative cases, (*g*) number of people receiving hospital treatment for COVID-19 and (*h*) cumulative COVID-19 deaths. Blue curves show the median of 500 model simulations and shaded bands show the 5th, 25th, 75th and 95th percentiles. Purple curves/points show data available at 7 July 2022 that was used to fit the model; green curves/points show subsequent validation data. Data shown in (*b*–*d*) are a 7-day rolling average. Data for new daily infections per 100 000 in (*a*) show the rate of cases detected in a routinely tested cohort of border workers. Reported cases are lower than total infections due to under-reporting. The most recent 10 days of deaths data and 40 days of admissions data are excluded due to reporting lags. Note: hospital occupancy in (*g*) is lower than number of COVID-19 cases in hospital as reported in the Ministry of Health’s daily updates, which includes some patients who are not being treated for COVID-19.

**Figure 4.**
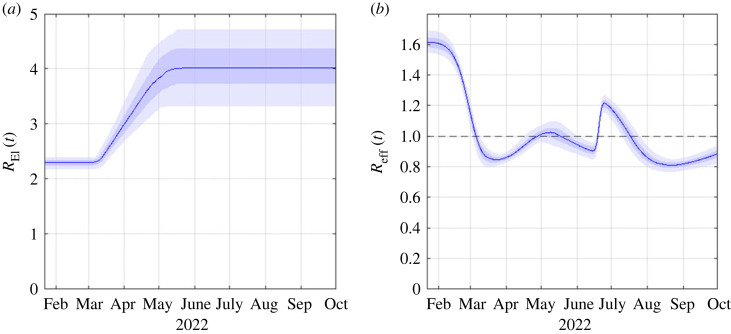
(*a*) Reproduction number excluding immunity *R*_EI_(*t*) and (*b*) effective time-varying reproduction number *R*_eff_(*t*). Blue curves show the median of 500 model simulations and shaded bands show the 5th, 25th, 75th and 95th percentiles. *R*_EI_(*t*) represents the potential spread rate in a fully susceptible population and changes in *R*_EI_(*t*) represent changes in contacts between individuals, as a result of changing public health measures and/or voluntary behaviour. *R*_eff_(*t*) represents the instantaneous reproduction number at time *t* including the effects of immunity. When *R*_eff_(*t*) > 1, the number of new infections increases with time, and when *R*_eff_(*t*) < 1, the number of new infections is decreasing with time.

Figure 3 shows model results for a scenario in which BA.5 has a growth rate of $0.09\;{\mathrm{day}}^{-1}$ relative to BA.2 and assuming that contact rates and government policy do not change in response to the wave. The growth advantage of BA.5 was estimated from data on sequenced community cases up to 21 June 2022 [24] (see §2). Scenarios where BA.5 has a smaller or larger growth advantage are shown in electronic supplementary material, figures S8 and S9. The results in figure 3 provide a reasonably good fit to the historic time series (purple curves and points in figure 3) from the start of the first Omicron wave in February 2022 through to June 2022. The long tail and relatively flat plateau between April and June 2022 can be explained by a gradual increase in the reproduction number excluding immunity *R*_EI_(*t*) in the model (see figure 4) and relaxation of the contact matrix describing mixing between age groups towards pre-pandemic patterns. This can be interpreted as a gradual relaxation of public health measures (e.g. shortening of isolation period, narrowing definition of close contact, removal of gathering size limits and mask mandates in schools) and of voluntary risk-reduction behaviours between March and May 2022.

Comparing with subsequently available data (green curves and points in figure 3), the peak in daily cases was slightly lower and occurred slightly earlier than the model predicted, although the trajectory remained within the 50% credible interval (CrI) of the model until after the peak, and within the 90% CrI (figure 3*b*) for the whole period. The peak in new daily hospital admissions (figure 3*c*) and hospital occupancy (figure 3g) were very close to model predictions. The model underestimated the number of COVID-19 deaths, although again the trajectory for daily deaths was within the 90% CrI of the model (figure 3*d*) and cumulative deaths were at the upper end of the 50% CrI (figure 3*h*). Notably, as predicted by the model, hospital occupancy and deaths were significantly higher in the July BA.5 wave than in the March BA.1/2 wave. Although new hospital admissions peaked at a similar level of around 100 per day in both waves, hospital occupancy peaked at around 500 in March and around 700 in July 2022. This is largely due to the fact that the average length of hospital stay increases with age (see electronic supplementary material, table S2). Similarly, deaths attributed to COVID-19 peaked at a 7-day rolling average of around 15 per day in March and 25 per day in July 2022.

**Figure 5.**
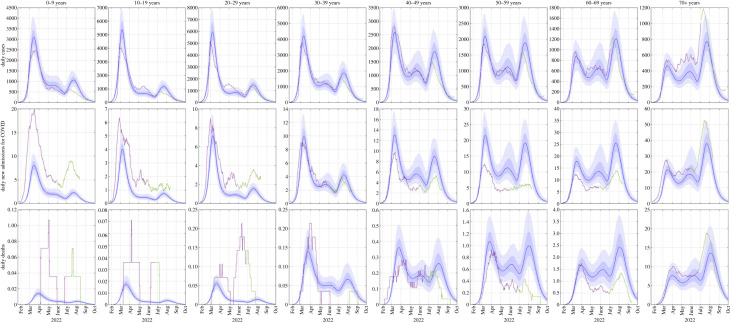
Age-stratified results showing new daily cases, new daily hospital admissions and daily deaths. Blue curve shows the median of 500 model simulations and shaded bands show the 5th, 25th, 75th and 95th percentiles. Purple curves show data available at 7 July 2022, and green curves show subsequent validation data. Data are shown as a rolling average over 7 days for cases, 14 days for admissions and 28 days for deaths. Note different *y*-axis scales in different plots due to large differences among age groups.

Stratifying cases, hospital admissions and deaths by 10-year age groups (figure 5) shows that the model provides a reasonable although imperfect representation of the age gradients in risk of infection and severe disease, and how these have changed over time. The first Omicron wave in March 2022 was dominated by younger age groups, particularly 10- to 30-year-olds. However, the age breakdown of cases has steadily shifted into older age groups over time (see electronic supplementary material, figure S6) and the second wave (BA.5) was larger than the first wave (BA.2) in over-60-year-olds. The model captures this shift, although it underestimated the size of the BA.5 peak in over-70-year-olds, which is likely to be a key driver of the high number of deaths relative to the number of cases. The model also underestimated hospital admissions and deaths in under-30-year-olds and overestimated them in 50- to 70-year-olds.

**Figure 6.**
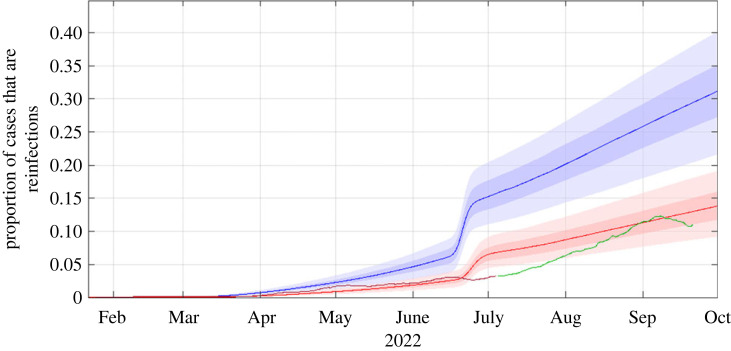
Proportion of reported cases that are reinfections. Model results show the true proportion of new reported cases that are reinfections (blue curve and bands) and the proportion adjusted for under-ascertainment of the first infection (red curve and bands). Curves shows the median of 500 model simulations and shaded bands show the 5th, 25th, 75th and 95th percentiles. Data available on 7 July 2022 (purple curve) and after 7 July 2022 (green curve) show the 7-day rolling average number of new cases that have previously reported a positive SARS-CoV-2 test at least 28 days prior as a fraction of the 7-day rolling average of total new cases.

Figure 6 shows the proportion of new reported cases that are reinfections. In the model, the true proportion of cases that are reinfections (blue curves in figure 6) increased from around 5% prior at the start of June 2022 to around 30% by October 2022. The rapid increase in reinfections seen in late June 2022 in the model is due to the greater ability of the BA.5 subvariant to evade immunity from prior infection with BA.2. In reality, cases can only be classified as reinfections if both the first and second infections were confirmed and reported to the Ministry of Health. Adjusting for under-ascertainment of first infections according to the age-specific case ascertainment ratio in the model results in a lower estimated reinfection rate (red curves in figure 6). This agrees more closely with empirical observations, although the increase in reinfections occurred more gradually than the model predicted.

## 4. Discussion

We have presented a mathematical model for SARS-CoV-2 in New Zealand since the arrival of Omicron in January 2022 that was used to estimate the impact of the recent BA.5 wave. The model combines previous work modelling COVID-19 dynamics in the New Zealand population [23], a published model for the relationship between neutralizing antibody titres and immunity to different outcomes [26,27] and the addition of a novel model component for the replacement of a resident variant with a fitter variant. Our results showed that the model made reasonable projections of the BA.5 wave in July and August 2022.

Strengths of the model include that it is fitted to five separate data series spanning a five-month period: daily COVID-19 cases, hospital admissions and deaths, proportion of new cases in over-60-year-olds and new weekly infections in a routinely tested cohort. The model strikes a balance between including sufficient complexity to capture the most important mechanisms affecting epidemic dynamics and explaining historical patterns, while remaining simple enough that results can be explained and understood with reference to particular assumptions. This is important for communicating results to policymakers and practitioners and avoids having a large number of unknown parameters and/or overfitting.

The model for the effect of a newly dominant variant is a simplification that produces an increase in epidemic growth rate by an increase in the susceptibility of the vaccinated population and the population previously infected with a different variant. The relationship of immunity with average neutralizing antibody titres means that the size of the increase in susceptibility is captured by a small number of model parameters. This has the advantage of avoiding the complexities of explicitly modelling simultaneous transmission of two strains with partial cross-immunity [33], while enabling real-time estimates of the parameters quantifying the new variant’s fitness advantage to be made from genomic data [24]. In this study, we used this approach to model the displacement of BA.2 by BA.5. However, it could be used in future to model similar displacements by new variants with varying characteristics and fitness advantages.

The model has a number of limitations and sources of uncertainty. The model underestimated the size of the BA.5 wave peak in over-70-year-olds. The model also underestimated hospital admissions and deaths in under-30-year-olds and overestimated them in 50- to 70-year-olds. There are several possible explanations for these discrepancies. The relaxation of the contact matrix describing mixing between age groups towards pre-pandemic patterns may not have adequately captured the increase in contacts with older age groups between the BA.2 wave in March 2022 and the BA.5 wave in July 2022. Immunity may be weaker and wane more rapidly in older people, as suggested by some studies, Bajaj *et al.* [38] and Müller *et al.* [39]. Or the assumed shape of the infection hospitalization ratio and infection fatality ratio [40] may be slightly mis-specified. The fact that the model groups everyone aged over 75 years into a single age band may also be contributing to systematically underestimating severe health outcomes, as these will be sensitive to the age distribution of cases within this group. Comparisons may also be affected by the fact that cause-of-death data are still not available for approximately 10% of deaths that have occurred within 28 days of a positive COVID-19 test.

Model predictions for the proportion of cases that are reinfections were largely consistent with available data after adjusting for under-ascertainment of first infections. This comparison is subject to several biases and should be viewed as approximate. For example, the adjustment of model output for under-ascertainment of first infections assumes that reporting of first and subsequent reinfections occur independently with the same probability. Data on reinfections could be biased upwards if there is individual-level heterogeneity in propensity to report an infection that persists through time, or biased downwards if people are less likely to test or report a second infection. It is also possible that in some cases the second positive test result could represent a chronic infection rather than a reinfection.

We have fitted the model to data using relatively informative priors for selected parameters. This provides a range of parameter combinations which are consistent with empirical data, but it cannot rule out that other parameter combinations or other sets of model assumptions could provide an equally good fit. This means that the uncertainty bands plotted in model results probably underestimate true uncertainty, especially when projecting model dynamics a long way into the future.

Reported cases are likely to be a significant underestimate of total infections, and the number of people infected with Omicron to date is unknown. Although we have used data from a routinely tested cohort of border workers, this cohort may not be representative of the population. This means the extent of infection-derived immunity in the population is uncertain. This uncertainty could be overcome in future by regular testing of a representative sample for SARS-CoV-2 [41,42].

Unlike previous waves of SARS-CoV-2 in New Zealand, immunity is now the single biggest factor affecting transmission dynamics. The immune landscape has become more complex over time, with various combinations of immunity derived from vaccination and prior infection with different subvariants at different time points. The model necessarily made simplifying assumptions about the nature of the immunity landscape, and it is possible that results are sensitive to these assumptions. For example, there are biological and epidemiological data [38,39] that suggest that vaccine-derived or infection-derived immunity could be lower and wane faster in older age groups or immunocompromised people. However, the data to parametrize an age-dependent immunity model are limited, and so we made the simplifying assumption that immunity provides an age-independent reduction in risk. In addition, we simplified the immunity model to avoid having to include compartments for too many different combinations and permutations of immunogenic events. This model structure meant that someone who was infected and then received their first or second vaccine dose had a higher level of immunity than someone who had one or two vaccine doses and was then infected.

Estimates for the level of immune escape of BA.5 are subject to uncertainty, and this was a key factor determining the size of the wave. We estimated the growth advantage of BA.5 relative to BA.2 from data on sequenced community cases reported up to 21 June 2022 [24]. Although this gave an estimate which was consistent with international estimates [10], it should be remembered that sequenced cases are not necessarily representative of all cases, and there is still a range of possible values for model parameters relating to the characteristics of BA.5. The effect of immune escape and reinfection on the risk of severe disease and death is also uncertain, and this could have affected the projected case hospitalization ratio and case fatality ratio.

The extent, if any, of behavioural change in response to a SARS-CoV-2 wave is difficult to predict in advance. In the model, we assumed a gradual increase in the reproduction number excluding immunity *R*_EI_(*t*) between March and May 2022 which can be interpreted as a gradual relaxation of public health measures and of voluntary risk-reduction behaviours. A gradual increase in *R*_EI_(*t*) over a period of time rather than step increases on dates associated with specific policy changes is consistent with observations from the relaxation of public health restrictions in England in 2021 [16]. Although behavioural change is known to have had a significant effect on previous waves in New Zealand, and internationally, it could not be assumed that the response would be comparable for the BA.5 wave or future waves. The model assumed that mixing within and between age groups could be reasonably approximated by an age-structured contact matrix. It is likely that population heterogeneity not accounted for in the model has a significant effect on the point at which new infections peak and start to decline. The size and timing of peaks are inherently uncertain because they are sensitive to variables and parameters that are not precisely known, including those mentioned earlier.

We only modelled the impacts of the BA.5 wave at the aggregate national level. These results will mask significant regional and demographic variation. Some groups are likely to be disproportionately affected, such as those working in public-facing roles and insecure employment, people in overcrowded or substandard housing, Māori and Pacific people [43,44] and people without good access to healthcare, testing, masks and vaccines [45].

## Data Availability

Data and Matlab code to reproduce the analysis are available at https://github.com/michaelplanknz/modelling-ba5-in-nz and as electronic supplementary material. The data are provided in electronic supplementary material [46].
